# SA-TD3: UAV-assisted task offloading and optimized resource allocation for maritime edge computing

**DOI:** 10.1038/s41598-025-31056-4

**Published:** 2025-12-02

**Authors:** Dechuan Kong, Shuhang Li, Yandi Zhang, Xiaohu Zhao, Yanyan Wang, Hongzhou Qiu, Yuhang Li, Wangyang He

**Affiliations:** 1 School of Artificial Intelligence, Henan Institute of Science and Technology, 453003 Xinxiang, China; 2 National and Local Joint Engineering Laboratory of Internet Application Technology on Mine, China University of Mining and Technology, 221116 Xuzhou, China; 3 School of Information Science and Engineering, Shenyang University of Technology, 110870 Shenyang, China

**Keywords:** UAV-Assisted task offloading, Maritime edge computing, Simulated annealing algorithm, TD3, Engineering, Mathematics and computing

## Abstract

In the large-scale Marine Internet of Things, UAV-assisted task offloading as a promising solution alleviates the computational burden encountered by fixed maritime surface nodes in multi-task ocean scenarios. However, most existing works focus on UAV trajectory design and resource allocation, while overlooking the dynamic demands and optimization potential of maritime surface nodes. Moreover, static resource allocation strategies and single optimization methods often limit global search capability and adaptability in dynamic ocean environments. We propose SA-TD3, a hybrid decision-making framework. We design a UAV-assisted computation offloading and resource optimization mechanism from the perspective of maritime surface nodes to better capture dynamic task demands. Furthermore, we develop an enhanced TD3 algorithm that integrates simulated annealing with an environment-aware dual-channel advantage function, improving global search capability and policy robustness. Finally, we construct a graph neural network-based dynamic prioritized replay mechanism to capture inter-node correlations and improve training efficiency. Extensive experiments demonstrate that SA-TD3 reduces average latency by 19.7% and improves overall performance by 13.2% across diverse ocean environments, effectively reducing the computational load and communication latency of surface nodes while enhancing energy efficiency.

## Introduction

Recently, the rapid advancement of maritime transportation, subsea exploration and development, and marine rescue missions has positioned the marine economy as a critical driver of global growth^1–3^. Within this context, the integration of autonomous underwater vehicles (AUVs) and the Marine Internet of Things (MIoT) has become a cornerstone of marine informatization. AUVs, owing to their high maneuverability, autonomous navigation, and adaptability to harsh environments, have emerged as indispensable tools for underwater exploration and operations^4^. They are widely deployed in tasks such as seabed topography mapping, mineral resource exploration, and marine ecological monitoring, while simultaneously collecting large volumes of real-time data. These data are transmitted to maritime surface nodes for storage, processing, and analysis, which support decision-making and subsequent mission planning. As critical components of MIoT, maritime surface nodes serve as intermediaries for AUV data transmission and integrate multi-source information collected by various maritime surface sensors, forming a comprehensive ocean information sensing network^5^.

However, maritime surface nodes usually possess limited computing and storage capabilities. In complex scenarios involving multiple concurrent tasks, it frequently leads to computational bottlenecks and degraded system performance. The issue is aggravated as the scale of marine monitoring expands, since the exponential growth of processed data exacerbates the computational burden on maritime surface nodes. Moreover, data transmission across seawater suffers from severe attenuation, while oceanic turbulence and varying sea states introduce unpredictable interference. These factors hinder the ability of maritime surface nodes to efficiently handle real-time data processing and transmission, thereby restricting their adaptability to dynamic ocean requirements^6–8^.

The emergence of unmanned aerial vehicles (UAVs) offers new opportunities to alleviate the computational and communication burden of maritime surface nodes^9^. UAVs, with their flexibility, wide coverage, and rapid deployment, can serve as mobile base stations or airborne edge servers to provide on-demand computational support^10,11^. Cooperative task processing between UAVs and maritime surface nodes can optimize resource allocation, reduce transmission latency, and enhance overall system performance^12,13^.

Early UAV-assisted computation offloading approaches primarily relied on mathematical modeling and optimization techniques such as convex optimization, integer linear programming, and heuristic algorithms^14–18^. For instance, Liu et al.^19^ improve system efficiency via a hybrid genetic algorithm for task offloading. Guan^20^ investigates a multi-priority allocation algorithm based on network topology and preference ordering. Sun et al.^21^ proposed a joint optimization of task offloading, computation resource allocation, and UAV trajectory control in multi-UAV-assisted MEC systems. Xu et al.^22^ addressed UAV-assisted MEC in Marine IoT by jointly optimizing user scheduling, UAV trajectory, and resource allocation. Zeng et al.^23^ investigated USV-assisted maritime wireless networks by jointly optimizing trajectory and resource allocation. These methods are capable of yielding near-optimal solutions in small-scale or static scenarios, offering clear theoretical interpretability and controllable optimization objectives. However, they usually assume homogeneous or static node capabilities and fail to capture the dynamic workload variations, heterogeneous resources, and adaptive offloading demands of maritime surface nodes. Consequently, they lack scalability in complex, time-varying environments where maritime surface nodes play a critical role in computation and communication^24^.

With the development of artificial intelligence (AI), deep reinforcement learning (DRL) has become a powerful paradigm for UAV-assisted task offloading and resource allocation^25^. Unlike traditional optimization approaches, DRL learns adaptive policies through continuous interaction with dynamic environments, making it particularly suitable for handling the complexity, heterogeneity, and uncertainty of MIoT^26–28^. Recent works have applied DRL to various aspects of maritime resource management. Luo et al.^29^ proposed DERLOC, a DRL-based solution using SAC and drift prediction to enhance the reliability of UAV–buoy optical communications. Fu et al.^30^ designed a cooperative relay mechanism for maritime data collection, integrating adaptive partitioning, game-theoretic routing, and MADDPG-based path planning. Tesfaw et al.^31^ developed an integrated LEO satellite and multi-UAV MIoT architecture with GMM-based clustering and FL-MADDPG for joint trajectory and resource allocation. Wu et al.^32^ introduced PG-MAPPO, which combines population-based learning and GMM adjustment for efficient UAV swarm task allocation. Akter et al.^33^ addressed task offloading and resource allocation in UAV mother-ship edge computing, proposing the SAC-based SATORA algorithm that considers energy consumption, latency, and resource constraints, enabling CPU-GPU co-execution and adaptation to dynamic environments.

In addition, a hybrid strategy that integrates reinforcement learning with traditional combinatorial optimization methods can ensure solution feasibility and stability, while simultaneously enhancing system adaptability and efficiency in dynamic environments. Hu et al.^34^ proposed the DGTT algorithm, based on deep reinforcement learning and game theory, to optimize UAV trajectories, pricing strategies, and user offloading decisions, thereby minimizing delay and energy consumption while enhancing the utilities of both UAVs and users. Hao et al.^35^ combined dependence-aware latent-space representation and deep reinforcement learning to jointly optimize UAV trajectories, offloading decisions, and transmission power, enhancing long-term task success rate. Zhang et al.^36^ proposed the EP-MUSTO algorithm based on digital twins and entropy-enhanced PPO, jointly optimizing delay, energy consumption, and encryption costs for efficient multi-UAV collaborative offloading.

Although these studies demonstrate the promise of DRL, several challenges remain. First, DRL algorithms often converge slowly and are prone to local optima, limiting their applicability in large-scale, real-time maritime scenarios. Second, most works adopt a UAV-centric optimization perspective, focusing on trajectory, transmission power, or offloading ratios, while neglecting the heterogeneous computational demands and real-time status of maritime surface nodes, resulting in inefficient resource utilization^37–40^. Finally, current approaches generally lack mechanisms for dynamic multidimensional resource allocation, as they fail to jointly consider computation, communication, and energy efficiency under varying conditions.

To address the above limitations, we propose SA-TD3, a hybrid decision-making framework for UAV-assisted task offloading and resource allocation in MIoT. By jointly considering the computational capacity, workload, and network status of surface nodes, SA-TD3 addresses the limitations of UAV-centric optimization and static allocation strategies, enabling adaptive offloading, cooperative UAV–node collaboration, and dynamic multidimensional resource management.

The main work of the article can be summarized as follows.

1. We propose SA-TD3, a hybrid decision-making framework that incorporates a UAV-assisted computation offloading and resource optimization mechanism from the perspective of maritime surface nodes, aiming to better address dynamic task demands in multi-task maritime scenarios.
2. We design an enhanced TD3 algorithm by integrating simulated annealing with an environment-aware dual-channel advantage function, thereby improving global search capability and policy robustness.
3. We construct a graph neural network-based dynamic prioritized replay mechanism to capture inter-node correlations and enhance training efficiency.
4. Simulations validate the superiority of the SA-TD3 in reducing average latency, alleviating computational load, optimizing energy efficiency, and improving overall system performance.

The rest of the article is organized as follows: Section “System model” describes the system model. Section "Hybrid decision-making framework" introduces the SA-TD3 algorithm. Section "Experiments and analysis" presents the experimental results and analysis, and Section “Conclusion” concludes the work.

## System model

This section formulates the system model for UAV-assisted task offloading in MIoT, focusing on computational requirements, resource allocation objectives, and task dependency constraints. We define the state space, action space, reward function, and transition probabilities, which provide the theoretical foundation for algorithm design.

### Maritime surface model

**Fig. 1:**
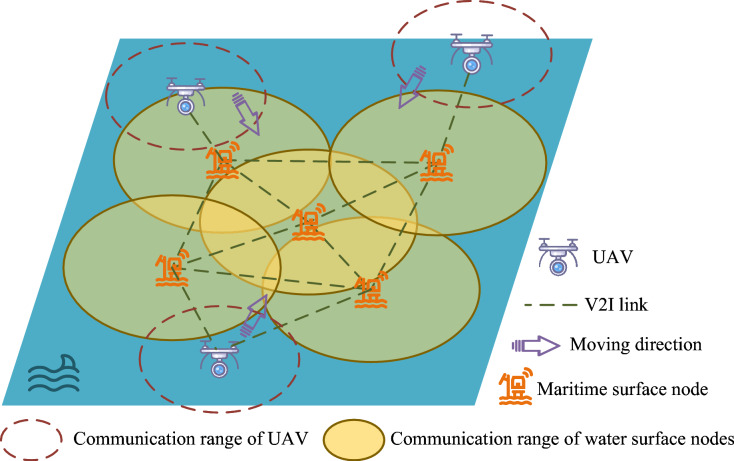
Maritime surface model.

As shown in Fig. 1, the system comprises maritime surface nodes and a UAV operating at a fixed altitude, *h*. Maritime surface nodes are randomly distributed over the ocean surface and are responsible for processing data collected from underwater devices. When resource overload occurs, resource overload nodes serve as pending-service nodes, while nodes that accept and execute offloaded tasks serve as task nodes or candidate nodes. Pending-service nodes mitigate their computational burden by offloading tasks to UAVs and nearby candidate nodes. These tasks may exhibit interdependencies, where the completion of one task relies on the prior execution of another.

Communication between surface nodes and the UAV is established through Vehicle-to-Infrastructure (V2I) links, based on Cellular Vehicle-to-Everything (C-V2X) technology. Due to the impact of sea states and seawater attenuation, the transmission efficiency between UAVs and maritime surface nodes is higher than that of communication between surface nodes. Furthermore, UAVs possess stronger computational capacity, enabling them to process larger workloads compared to individual maritime surface nodes.

In this model, the set of pending-service nodes at a given moment *t* is defined as $Z_{n}=\{z_{1},z_{2},...,z_{n}\}$, each associated with one task $M_{n}={m_{1},m_{2},...,m_{n}}$. A task $m_{n}$ is defined by a triplet $m_{n}={D_{n},X_{n},T_{n}^{\textrm{max}}}$, where $D_{n}$ is the data size, $X_{n}$ is the required computational resources, and $T_{n}^{\textrm{max}}$ is the maximum tolerable delay.

For a divisible task $m_{n}$, it can be represented as a set of subtasks $A_{n}=\{a_{(n,1)},a_{(n,2)},...,a_{(n,z)}\}$, where $a_{(n,z)}$ denotes the *z*-th subtask of the *n*-th task. Each subtask $a_{(n,z)}$ is similarly defined by a triplet $a_{(n,z)}=\{d_{(n,z)},x_{(n,z)},d_{(n,z)}^{\textrm{max}}\}$, in which $d_{(n,z)}$ is the data volume, $x_{(n,z)}$ is the computational resource demand, and $d_{(n,z)}^{\textrm{max}}$ is the maximum tolerable delay.

### Task dependency model

**Fig. 2:**
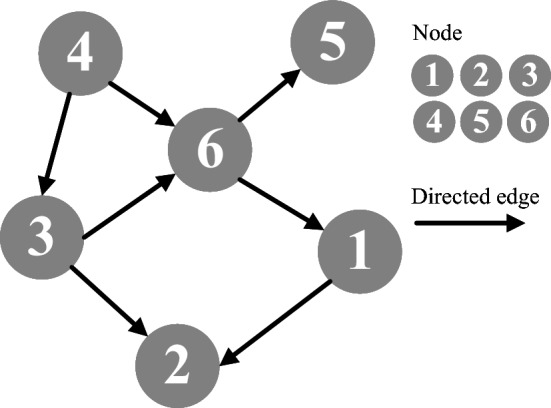
Directed acyclic graph.

A directed acyclic graph (DAG) is a specialized graph structure consisting of nodes and directed edges, with the defining characteristic that it contains no cycles. In other words, starting from any node and following the edge directions, it is impossible to return to the same node. This property makes DAGs particularly suitable for representing task sequences and dependencies, as they naturally capture the logical order of task execution^41^.

In this model, a dependent task is expressed as $Y_{n} = {A_{n}, H_{n}}$, where $A_{n}$ is the subtask set of task *n* and $H_{n}$ represents dependency edges. For a given edge ${A_{(n,i)},A_{(n,j)}} \in H_{n}$, subtask $A_{(n,j)}$ depends on $A_{(n,i)}$, meaning that $A_{(n,i)}$ must be completed before $A_{(n,j)}$. Thus, $A_{(n,i)}$ is the predecessor and $A_{(n,j)}$ the successor.

Specifically, a subtask without any predecessors is called an entry task, while a subtask without any successors is called an exit task. Figure 2 illustrates a pending task $M_{n}$. Task 4 is an entry task, tasks 2 and 5 are exit tasks, task 3 is the predecessor of tasks 6 and 2, and task 6 is the successor of tasks 3 and 4.

### Transmission and calculation model

#### Local model

When a subtask $a_{(n,z)}$ is executed locally at a pending-service node $z_{n}$, transmission delay is negligible. Therefore, the total delay $T_{(n,z)}$ consists solely of the local computation delay $t_{(n, z)}^{\textrm{comp}}$. Accordingly, the computation delay for processing subtask $a_{(n,z)}$ of task $m_{n}$ at pending-service node $z_{n}$ is expressed as,

1 $$\begin{aligned} T_{(n,z)} = t_{(n,z)}^{\textrm{comp}}=\frac{x_{n,z}}{f_{(n,z)}} \end{aligned}$$

where $x_{n,z}$ is the required computational workload and $f_{(n,z)}$ is the available computational capacity of node $z_{n}$.

#### Maritime surface node model

Pending-service nodes may offload subtasks to nearby candidate nodes via C-V2X-based V2I communication. Assuming no other factors affect connections between them, the transmission rate between node $z_{n}$ and candidate node *c* is expressed as,

2 $$\begin{aligned} r_{(z_{n},c)}=W_{(z_{n},c)}log_{2}(1+\frac{p_{(z_{n},c)}h_{(z_{n},c)}}{\sigma ^{2}l_{(z_{n},c)}}), \end{aligned}$$

where $W_{(z_{n},c)}$ is the allocated bandwidth, $p_{(z_{n},c)}$ is the transmission power, $h_{(z_{n},c)}$ is the channel gain, and $\sigma ^{2}$ is the noise power. $l_{(z_{n},c)}$ is the Euclidean distance from the pending service node $z_{n}$ to the other idle nodes *c*, which is expressed as,

3 $$\begin{aligned} l_{(z_{n},c)}=\sqrt{(x_{z_{n}}-x_{c})^{2}+(y_{z_{n}}-y_{c})^{2}}. \end{aligned}$$

The delay $T_{(z_{n},c)}$ incurred when processing a task at surrounding idle surface nodes consists of two parts: the transmission delay $t_{(z_{n},c)}^{\textrm{tran}}$, which corresponds to the time required for the pending-service node to upload task data to the idle node, and the computation delay $t_{(z_{n},c)}^{\textrm{comp}}$, which corresponds to the time required for the task node to process the received task. The overall delay can be expressed as,

4 $$\begin{aligned} T_{(z_{n},c)}=t_{(z_{n},c)}^{\textrm{tran}}+t_{(z_{n},c)}^{\textrm{comp}}, \end{aligned}$$

where $t_{(z_{n},c)}^{\textrm{tran}}$ and $t_{(z_{n},c)}^{\textrm{comp}}$ can be expressed as,

5 $$\begin{aligned} t_{(z_{n},c)}^{\textrm{tran}} = \frac{d_{(n,z)}}{r_{(z_{n},c)}}, \end{aligned}$$

6 $$\begin{aligned} t_{(z_{n},c)}^{\textrm{comp}}=\frac{x_{(z_{n},c)}}{f_{(z_{n},c)}}, \end{aligned}$$

where $f_{(z_{n},c)}$ represents the computing resources of the offloaded maritime surface node device.

#### UAV model

Assuming wireless communication between the maritime surface node and the UAV occurs via the C-V2X-based V2I transmission mode without additional interference factors, the transmission rate between the pending-service node and the UAV is expressed as,

7 $$\begin{aligned} r_{(z_{n},f)}=W_{(z_{n},f)}log_{2}(1+\frac{p_{(z_{n},f)}h_{(z_{n},f)}}{\sigma ^{2}l_{(z_{n},f)}}), \end{aligned}$$

where $W_{(z_{n},f)}$, $p_{(z_{n},f)}$, and $h_{(z_{n},f)}$ represent the communication bandwidth, transmission power, and channel gain between the pending-service node $z_{n}$ and the UAV *f*, respectively, $\sigma ^{2}$ is the noise power, which is the variance of Gaussian white noise, and $l_{(z_{n},f)}$ is the Euclidean distance from the pending-service node $z_{n}$ to the UAV *f*, which is expressed as,

8 $$\begin{aligned} l_{(z_{n},f)}=\sqrt{(x_{z_{n}}+x_{f})^2)+(y_{z_{n}}-y_{f})^2+(h_{z_{n}}-h_{f})^2}. \end{aligned}$$

The delay $T_{(z_{n},f)}$ incurred when offloading a task to UAV *f* consists of two components: the transmission delay $t_{(z_{n},f)}^{\textrm{tran}}$, which corresponds to the time required for the pending-service node to upload task data to UAV *f*, and the computation delay $t_{(z_{n},f)}^{\textrm{comp}}$, which corresponds to the time required for UAV *f* to process the received data. The overall delay can be expressed as,

9 $$\begin{aligned} T_{(z_{n},f)}=t_{(z_{n},f)}^{\textrm{tran}}+t_{(z_{n},f)}^{\textrm{comp}}, \end{aligned}$$

where $t_{(z_{n},f)}^{\textrm{tran}}$ and $t_{(z_{n},f)}^{\textrm{comp}}$ can be expressed as,

10 $$\begin{aligned} t_{(z_{n},f)}^{\textrm{tran}} = \frac{d_{(n,z)}}{r_{(z_{n},f)}}, \end{aligned}$$

11 $$\begin{aligned} t_{(z_{n},f)}^{\textrm{comp}} = \frac{x_{z_{n}}}{f_{(z_{n},f)}}, \end{aligned}$$

where $f_{(z_{n},f)}$ represents the computing resources of the UAV to be offloaded, and $f_{(z_{n},f)}> f_{(z_{n},c)}$.

Additionally, at time *t*, the task $M_{n}$ of the pending-service node is partitioned into multiple subtasks, which are executed either on the node itself, nearby idle maritime surface nodes, or drones. The completion time of task $M_{n}$ is determined by the maximum delay among all its subtasks. Accordingly, the total delay for completing task $M_{n}$ at the pending-service node can be expressed as,

12 $$\begin{aligned} T=\sum _{n=1}^{N}\textrm{max}(T_{(n,z)},T_{(z_{n},c)},T_{(z_{n},f)}). \end{aligned}$$

### Energy consumption model

Maritime surface nodes and UAVs inevitably incur energy consumption during the processing of task data. To minimize total energy expenditure while ensuring that the execution delay does not exceed the maximum tolerable threshold, the energy consumption $E_{(n,z)}$ for local processing can be expressed as,

13 $$\begin{aligned} E_{(n,z)}=T_{(n,z)}P_{(n,z)}, \end{aligned}$$

where $P_{(n,z)}$ represents the power calculated locally by pending-service node $z_{n}$. Similarly, the energy consumption $E_{(z_{n},c)}$ of the surrounding idle nodes and $E_{(z_{n},f)}$ of the UAV can be expressed as,

14 $$\begin{aligned} E_{(z_{n},c)}=T_{(z_{n},c)}^{\textrm{comp}}P_{(z_{n},c)}, \end{aligned}$$

15 $$\begin{aligned} E_{(z_{n},f)}=T_{(z_{n},f)}^{\textrm{comp}}P_{(z_{n},f)}, \end{aligned}$$

where $P_{(z_{n},c)}$and $P_{(z_{n},f)}$ represent the power calculated by the surrounding idle nodes and the UAV, respectively.

The system objective is to minimize total energy consumption while ensuring that task execution delays remain within their maximum tolerable thresholds.

### Problem statement

In UAV-assisted MIoT, task dependencies are modeled using a Directed Acyclic Graph (DAG). Tasks with stringent latency or heavy computational requirements are assigned higher priority through topological sorting, ensuring that critical operations are executed promptly under limited resources. This mechanism allows the proposed SA-TD3 algorithm to manage complex dependencies, enhance responsiveness, and ensure stable operation in real-time maritime environments. The optimization objective is to jointly determine task offloading and resource allocation strategies that minimize both system-wide latency and energy consumption. The optimization problem can be formulated as,

16 $$\begin{aligned} \min _{a} \sum _{i=1}^{N}(\alpha \cdot T_{i}+\beta \cdot E_{i}), \end{aligned}$$

where *a* denotes the offloading decision vector for all tasks, *N* is the total number of maritime surface nodes, $T_{i}$ and $E_{i}$ represent the delay and energy consumption required for maritime surface node *i* to complete the task, respectively, and $\alpha$, $\beta$ are weights balancing the trade-off between latency and energy. The optimization problem provides the performance feedback that guides policy updates for SA-TD3.

The completion time of each task must be greater than the completion time of all its predecessor tasks plus communication and computation time. This constraint determines the state transition logic of the learning process. Only tasks whose predecessors have completed are considered available for offloading, ensuring that policy learning adheres to the DAG task dependencies. The constraint is expressed as,

17 $$\begin{aligned} T_{i} \ge T_{j}+T_{(i,j)}^{\textrm{tran}}, \end{aligned}$$

where $T_{i}$ is the follow-up task of $T_{j}$, and $T_{(i,j)}^{\textrm{tran}}$ is the communication and computation time from task *j* to task *i*.

Each node has limited computing resources, and the computing time of a task cannot exceed the maximum computing capacity of the node. This constraint defines the feasible action space for computing resource allocation. During policy exploration, any action exceeding maximum computing power is penalized through the reward function, guiding the agent toward efficient allocations. The constraint is expressed as,

18 $$\begin{aligned} \sum _{i=1}^{n}T_{i}\le R_{\textrm{max}}, \end{aligned}$$

where $R_{\textrm{max}}$ represents the maximum computing power of the node.

Finally, each node has limited communication bandwidth, and the communication data volume of the task cannot exceed the maximum communication bandwidth of the node. This constraint affects the transmission power and offloading decision actions of the SA-TD3. When an action leads to excessive bandwidth consumption, the SA-TD3 receives a negative reward, promoting learning of feasible transmission strategies within network capacity limits. The constraint is expressed as,

19 $$\begin{aligned} \sum _{i=1}^{n}D_{i}\le B_{\textrm{max}}, \end{aligned}$$

where $B_{\textrm{max}}$ represents the maximum communication bandwidth of the node.

Through the integration of these constraints, the SA-TD3 algorithm can learn a policy that operates within the feasible region of the optimization problem while achieving efficient trade-offs between latency and energy consumption.

## Hybrid decision-making framework

**Fig. 3:**
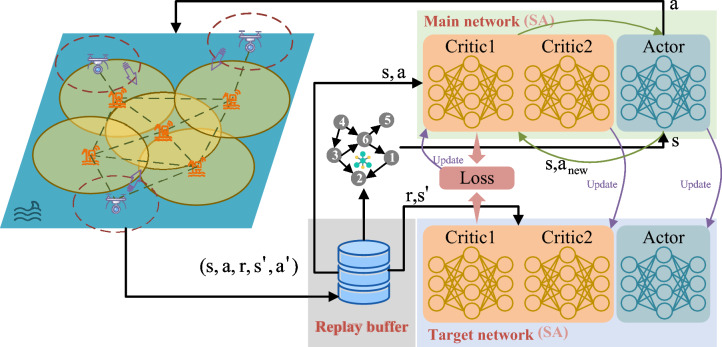
SA-TD3 offloading framework for maritime operations.

To address the computational overload problem at maritime surface nodes, this study proposes SA-TD3, a hybrid decision-making algorithm that combines deep reinforcement learning with global optimization. Specifically, a priority-based experience replay mechanism is incorporated into TD3 to enable more effective utilization of critical experiences, thereby accelerating convergence and improving learning efficiency. In addition, the integration of a value function refines the policy update process, leading to higher-quality decision-making. To further enhance global exploration capability and mitigate the risk of falling into local optima, simulated annealing (SA) is introduced into the task offloading decision process, enabling the identification of more optimal task allocation strategies.

Describe the computation offloading and resource allocation problem as a Markov Decision Process (MDP). An MDP can be represented by a quadruple $\{S, A, R\}$. The details of each element are as follows:

**State:** $S=\{ S_{t} \mid S_{t} = (D_{t},S_{t},T_{t}^{\textrm{max}},Q,f_{(n,z)},f_{(z_{n},c)},f_{(z_{n},f)})\}$, where each time slot *t* corresponds state $S_{t}$, $S_{t} \in S$. $D_{t}$, $S_{t}$, and $T_{t}^{max}$ represent the three attributes of the task, *Q* represents the task sorting result in the DAG, $f_{(n,z)}$ represents the locally available computing resources, $f_{(z_{n},f)}$ represents the available computing resources of the UAV, and $f_{(z_{n},c)}$ represents the available computing resources of the surrounding idle nodes.

**Action:** $A=\{ A_{t} \mid A_{t} = ( a_{1}, a_{2},...,a_{n})\}$, where each time slot t the agent sends the offloading decision. $a_{n}$ denotes the number of resource units allocated to the *n*-th task, and $a_{n}=0$ indicates that the task is computed locally. The size of the *n*-th task must not exceed the total computational resources *k* of the current computational unit.

**Reward:** The reward function of the system is expressed as,

20 $$\begin{aligned} \left\{ \begin{matrix} R_{t}=Klog_{2}(1+(t_{n}^{\textrm{max}}-t_{n}^{\textrm{total}}))-(E_{(n,z)}+E_{(z_{n},c)}+E_{(z_{n},f)}) & t_{n}^{\textrm{max}} \ge t_{n}^{\textrm{total}} \\ R_{t}=-1000 & t_{n}^{\textrm{max}} < t_{n}^{\textrm{total}} \end{matrix}\right. \end{aligned}$$

where $t_{n}^{\textrm{max}}$ represents the maximum delay that the current task can tolerate, and $t_{n}^{\textrm{total}}$ is the actual delay required to complete the current task. If $t_{n}^{\textrm{max}} \ge t_{n}^{\textrm{total}}$, then the required energy consumption is also considered in the reward function. If $t_{n}^{\textrm{max}} < t_{n}^{total}$, it indicates that the total delay of the current task exceeds the maximum tolerable delay, and a negative reward is given, $R_{t} = -1000$.

The SA-TD3 framework is shown in Fig. 3. It learns adaptive offloading and resource allocation policies that balance latency and energy efficiency while ensuring compliance with DAG-based task dependencies.

### Dynamic priority replay mechanism based on graph neural networks

The priority experience replay mechanism assigns a priority score to each experience stored in the replay buffer, ensuring that samples with higher importance are more likely to be selected during training^42^. This strategy improves the utilization of critical experiences and accelerates the convergence of the learning process. In the article, the priority weight $P_{i}$ of each experience is determined by integrating four-dimensional features, which is expressed as,

21 $$\begin{aligned} P_{i}=\alpha \cdot \textrm{Norm}(\tau _{i})+\beta \cdot \mathrm {GNN(DAG}_{i}) + \mu \cdot \beta _{(\textrm{TD},i)} + v \cdot f_{\textrm{decay}}(t-t_{i}), \end{aligned}$$

where $\tau _{i}$ denotes the remaining deadline ratio of the task, $\textrm{Norm}(\cdot )$ denotes the min–max normalization operator, $\mathrm {GNN(DAG}_{i})$ denotes the graph neural network embedding value extracted from the task dependency graph, which captures structural correlations among subtasks, $\beta _{(\textrm{TD},i)}$ corresponds to the absolute temporal-difference (TD) error, reflecting the learning value of the experience, and $f_{\textrm{decay}}$ denotes an exponential decay factor designed to suppress the influence of outdated samples.

When a new experience $e_{j}$ arrives, it is stored directly if the replay buffer is not yet full. Once the buffer reaches capacity, an elimination threshold $P_{\textrm{min}}$ is computed. If the priority of the new experience satisfies $P_{j} < P_{\textrm{min}}$, the sample with the lowest priority $e_{\textrm{min}}$ in the buffer is replaced. This ensures that the replay buffer continuously preserves experiences with higher contribution to training, thereby improving both sample efficiency and policy robustness.

### Environment-aware dual-channel advantage function

The advantage function is an extension of the action-value and state-value functions, which quantifies the relative benefit of taking a particular action compared with other possible actions under the same state. To better adapt to the characteristics of UAV-assisted maritime task offloading, we propose an environment-aware dual-channel advantage function, which simultaneously integrates the real-time load characteristics of the local execution scene and the topological constraints of the global environment. Formally, it is defined as,

22 $$\begin{aligned} A(s,a)=\lambda \cdot A_{\textrm{local}}(s_{d},a)+(1-\lambda ) \cdot A_{\textrm{global}}(s_{t},a), \end{aligned}$$

where $A_{\text {local}}(s_{d},a)$ captures the local computational load and resource utilization when action *a* is taken in dynamic scene parameter $s_{d}$, $A_{\text {global}}(s_{t},a)$ encodes the task-dependency and topological constraints derived from the DAG structure of the overall system, and $\lambda$ is weighting coefficients that balance local adaptability and global consistency.

This dual-channel design allows the policy network to optimize short-term resource allocation at individual nodes and maintain long-term consistency with task dependency constraints, thereby enhancing both local responsiveness and global coordination in dynamic maritime environments.

In reinforcement learning, directly optimizing the action-value function *Q*(*s*, *a*) often leads to high variance during training. The advantage function mitigates this issue by subtracting the state value from the action value, thereby stabilizing the learning process. However, in maritime environments, sudden fluctuations in ocean channels can cause rapid changes in advantage values, leading to instability. To address this, we introduce a sliding variance constraint into the output layer, expressed as,

23 $$\begin{aligned} \hat{A}(s,a)=\textrm{EMA}(A(s,a)) \cdot e^{-\frac{\sigma _{A}^{2}}{\gamma }}, \end{aligned}$$

where $\textrm{EMA}(\cdot )$ denotes an exponential moving average smoothing filter, $\sigma _{A}^{2}$ represents the variance of recent *k*-step advantage values, and $\gamma$ is a signal-to-noise-ratio–driven parameter that adaptively controls the decay.

Incorporating the environment-aware dual-channel advantage function with this variance-aware correction enhances the learning stability and adaptability of the algorithm. The dual-channel structure separates rapid environmental fluctuations from long-term value trends. The variance-based correction allows the update intensity to adjust automatically rather than relying on a fixed decay schedule. This combination prevents sluggish or overly rigid policy updates and enables the algorithm to track dynamic maritime communication conditions more effectively, leading to smoother optimization and improved robustness without introducing significant additional complexity.

### Algorithm

In the SA-TD3 algorithm, two complementary algorithms are jointly employed: the Simulated Annealing algorithm (SA) and the Twin Delayed Deep Deterministic Policy Gradient network (TD3).

The SA consists of state nodes representing system states during the annealing process, with probabilistic transitions governed by the Metropolis criterion. At each iteration, the system determines whether to accept a candidate state based on the current temperature and energy difference. The main parameters include the initial temperature $T_{0}$, the temperature decay rate $\alpha$, and the termination temperature $T_{\textrm{min}}$. These parameters control the search trajectory and ensure the ability to escape local optima. In the SA-TD3, SA serves as a global search component that enhances the network’s exploration capability. By probabilistically accepting worse solutions according to the Metropolis criterion, the algorithm can cross energy barriers in the optimization landscape and avoid premature convergence to suboptimal solutions. SA gradually decreases the temperature, which smoothly shifts the algorithm from global exploration to local exploitation, improving convergence stability.

During SA training, a new state $s_{\textrm{new}}$ is generated from the current state $s_{\textrm{current}}$. The energy difference is calculated as $\Delta E = E(s_{\textrm{new}}) - E(s_{\textrm{current}})$, where *E* is the energy function of the system. If $\Delta E \le 0$, the new state is accepted directly ($s_{\textrm{current}} = s_{\textrm{new}}$). Otherwise, $s_{\textrm{new}}$ is accepted with probability $p = e^{-\frac{\bigtriangleup E}{T}}$, where *T* is the current temperature. The process continues until $T < T_{\textrm{min}}$. This probabilistic acceptance mechanism enables the learning process to maintain sufficient diversity during the early training stage, helping the agent escape from locally optimal regions in the continuous state-action space.

The TD3 network is responsible for policy optimization in continuous state–action spaces. Its key parameters include the learning rate, experience replay buffer size, target network update frequency, and discount factor $\gamma$. These ensure stable convergence and efficient utilization of past experiences.

In the critic network update proceeds, given a mini-batch $(s_{i}, a_{i}, r_{i}, s_{i+1})$ sampled from the replay buffer, the target action is computed via the target actor network, which is expressed as,

24 $$\begin{aligned} a_{i+1} = \mu ^{'}(s_{i+1} \mid \theta ^{\mu ^{'}}). \end{aligned}$$

The target *Q*-value is estimated as,

25 $$\begin{aligned} y_{i}=r_{i}+\gamma \cdot minQ^{'}(s_{i+1},a_{i+1} \mid \theta ^{\mu ^{'}}). \end{aligned}$$

The critic parameters $\theta ^{Q}$ are updated by minimizing the Equation 25. The gradient calculation for $\theta ^{Q}$ is expressed as,

26 $$\begin{aligned} \bigtriangledown _{\theta ^{Q}}L=\frac{2}{N}\sum _{i}^{}(Q(s_{i},a_{i} \mid \theta ^{Q})-y^{i})\bigtriangledown _{\theta ^{Q}}Q(s_{i},a_{i}\mid \theta ^{Q}). \end{aligned}$$

The actor network parameters $\theta ^{\mu }$ are optimized to maximize the critic’s *Q*-value via gradient ascent, which is expressed as,

27 $$\begin{aligned} \theta ^{\mu }\leftarrow \theta ^{\mu } +\beta \bigtriangledown _{\theta ^{\mu }}J. \end{aligned}$$

Target networks are updated softly using parameter $\tau$, which is expressed as,

28 $$\begin{aligned} \theta ^{\mu ^{'}} \leftarrow \tau \theta ^{Q} + (1-\tau )\theta ^{\mu ^{'}} \end{aligned}$$

29 $$\begin{aligned} \theta ^{Q^{'}} \leftarrow \tau \theta ^{Q}+(1-\tau )\theta ^{Q^{'}} \end{aligned}$$

By combining TD3 with SA, the SA-TD3 framework achieves a better balance between exploration and exploitation. SA improves global exploration, while TD3 ensures local fine-tuning and stable policy improvement. This integration effectively accelerates convergence and enhances the robustness of policy learning in complex maritime environments. The SA-TD3 algorithm is summarized in Algorithm 1.

**Algorithm 1:**
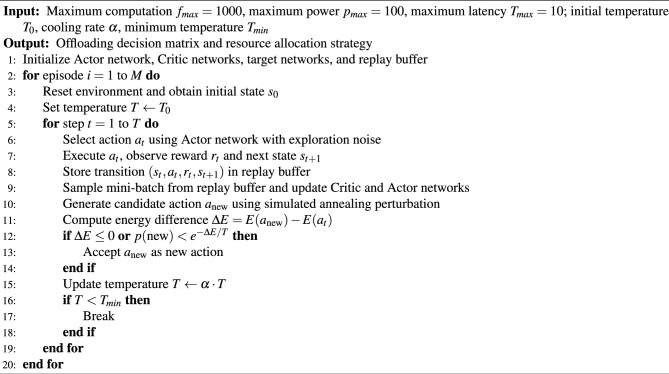
SA-TD3 Task Offloading Algorithm

**Table 1:** Key simulation parameters.

Parameters	Value	Unit
Number of water-surface nodes	$5 \sim 20$	-
Input data size	$200 \sim 1,200$	*KB*
Computational resources per task	100	-
Surface node computation capacity	1	$\text {GHz}\cdot s^{-1}$
UAV computation capacity	10	$\text {GHz}\cdot s^{-1}$
V2I communication bandwidth	1	*MHz*
Noise power	−114	*dBm*
Max. transmission power	23	*dBm*
UAV flight altitude	100	*m*
Transmission range	200	*m*
Max. tolerable delay	3	*s*
Task dependencies	DAG	-
Algorithm iterations	1,000	*times*
Learning rate	0.01	-
Discount factor	0.99	-
Replay buffer size	1,000	-
Target network update frequency	5	-

## Experiments and analysis

### Experimental design

To validate the effectiveness of the proposed UAV-assisted computation offloading and resource allocation framework, we conducted a series of simulation experiments in Python, with deep learning models implemented and trained using TensorFlow. The experimental setup emulates a MIoT environment where surface nodes are randomly distributed over the sea, while UAVs provide aerial computation offloading services. The design places particular emphasis on the interaction between maritime surface nodes and UAVs, reflecting the dynamic and heterogeneous characteristics of real-world maritime communication networks. The key simulation parameters are summarized in Table 1.

In the experiments, task dependencies were modeled using a directed acyclic graph (DAG), where each node represents a subtask and directed edges capture the dependency relationships between them. Subtask scheduling was performed dynamically based on both the DAG structure and the real-time computational states of surface nodes and UAVs. Depending on system conditions, each subtask could either be processed locally, migrated to nearby candidate surface nodes, or offloaded to UAVs for execution.

### Result analysis

Performance is compared against three baselines: fully local computation (ALL-local), fully edge offloading (ALL-edge), and the standard TD3 algorithm. The comparison focuses on three key performance metrics:

**Task completion rate**, measuring the proportion of tasks completed within their deadline.

**Average latency**, reflecting system responsiveness.

**Average reward**, representing overall efficiency in balancing latency and energy consumption.

**Fig. 4:**
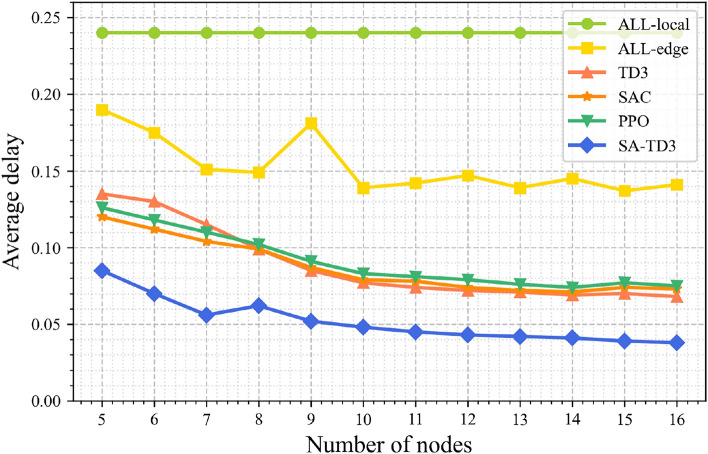
Average delay under different numbers of candidate nodes.

**Fig. 5:**
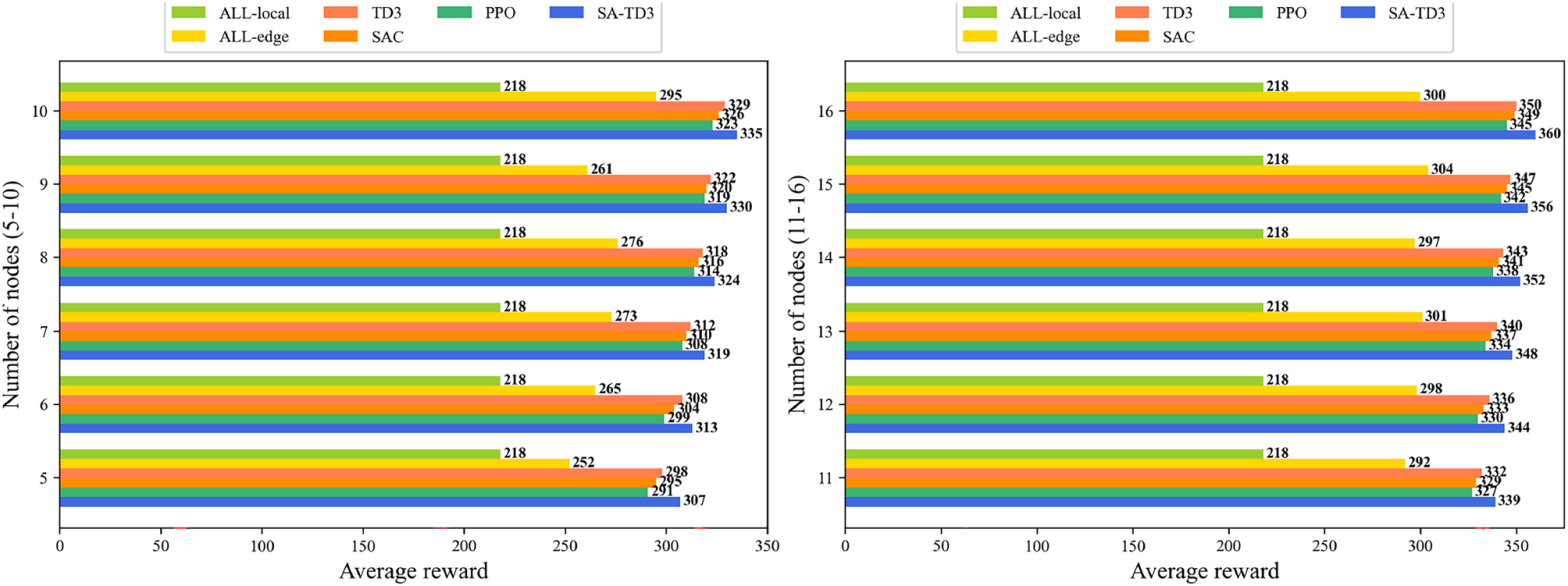
Average reward under different numbers of candidate nodes.

Figures 4 and 5 illustrate the performance of different algorithms under varying numbers of candidate maritime surface nodes, presenting average latency and average reward separately for clear comparison. As the number of candidate nodes increases from 5 to 16, all algorithms benefit from the additional computational resources, resulting in reduced latency and improved reward. SA-TD3 consistently achieves the lowest latency and highest average reward across all node counts, demonstrating its ability to efficiently leverage distributed resources and dynamically optimize task offloading.

In particular, the latency reduction achieved by SA-TD3 within the range of 5–16 idle nodes is more pronounced than that of the baseline algorithms. This result highlights the advantage of integrating simulated annealing with TD3, which enables better exploitation of idle node capacity, balanced workload distribution between local nodes and UAVs, and minimized communication and computation delays. For reward, SA-TD3 consistently delivers higher system returns across all tested conditions, reflecting superior resource utilization, energy efficiency, and responsiveness.

By contrast, the fully local computation scheme (ALL-local) shows persistently high latency and low reward regardless of node count, underscoring its inability to take advantage of additional idle resources. The fully edge offloading scheme (ALL-edge) achieves moderate improvements, but its reliance on UAV resources often leads to underutilization of local capacity, thereby limiting performance gains. Standard TD3 performs between these extremes, while it reduces latency and improves reward compared with ALL-local and ALL-edge, it remains less effective than SA-TD3, particularly in coordinating multiple idle nodes for global task optimization. SAC and PPO achieve lower delays and steadily increasing rewards, demonstrating their ability to adapt to increasing node densities. Among them, SAC performs marginally better than PPO and has better stability. Nonetheless, SA-TD3 consistently achieves the lowest delay across and the highest reward, demonstrating more efficient learning and resource allocation.

Overall, the combined analysis of latency and reward under varying candidate node counts shows that SA-TD3 consistently outperforms the baselines in responsiveness and efficiency, demonstrating good robustness and adaptability in resource-variable MIoT scenarios. Although extremely large-scale maritime networks with multiple UAVs are beyond the scope of our current simulation platform, the design of SA-TD3, particularly the augmented state representation and twin-critic structure, supports good scalability as the problem size grows. Moreover, scenarios with multi-node and multi-UAV cooperation remain important future extensions, as part of our future planned work.

**Fig. 6:**
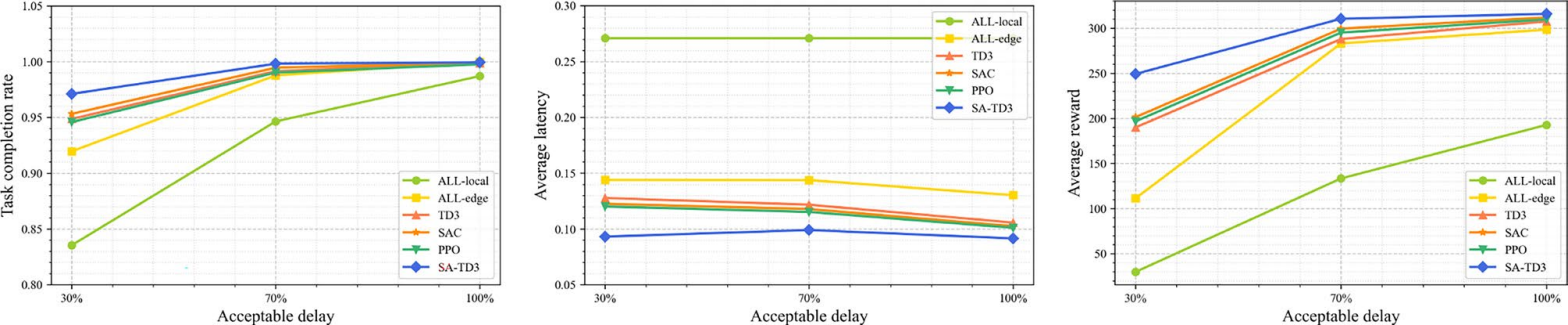
Task completion rate, average latency and reward under varying delay tolerances.

Figure 6 presents performance under varying delay tolerance thresholds, showing task completion rate, average latency, and average reward. Across all levels of latency tolerance, SA-TD3 consistently achieves the highest task completion rate, lowest latency, and highest reward, underscoring its adaptability to diverse real-time constraints.

SA-TD3 effectively balances offloading between local nodes, UAVs, and idle nodes, ensuring that delay-sensitive subtasks are prioritized and completed promptly. In contrast, ALL-local performs reasonably well only under relaxed latency requirements, but its completion rate and reward drop sharply under stricter constraints. ALL-edge demonstrates moderate improvements, but its inability to fully exploit local resources limits performance. Standard TD3 outperforms both ALL-local and ALL-edge yet fails to match the robustness of SA-TD3 when latency requirements are stringent. SAC and PPO improve task completion rate as delay tolerance increases, with SAC achieving lower latency than PPO due to its stochastic policy. However, SA-TD3 surpasses both methods across all metrics, maintaining the lowest latency, highest completion rate, and highest reward.

The above results show that SA-TD3 provides efficient task allocation and resource management across heterogeneous latency requirements, maintaining high system returns under strict real-time conditions.

**Fig. 7:**
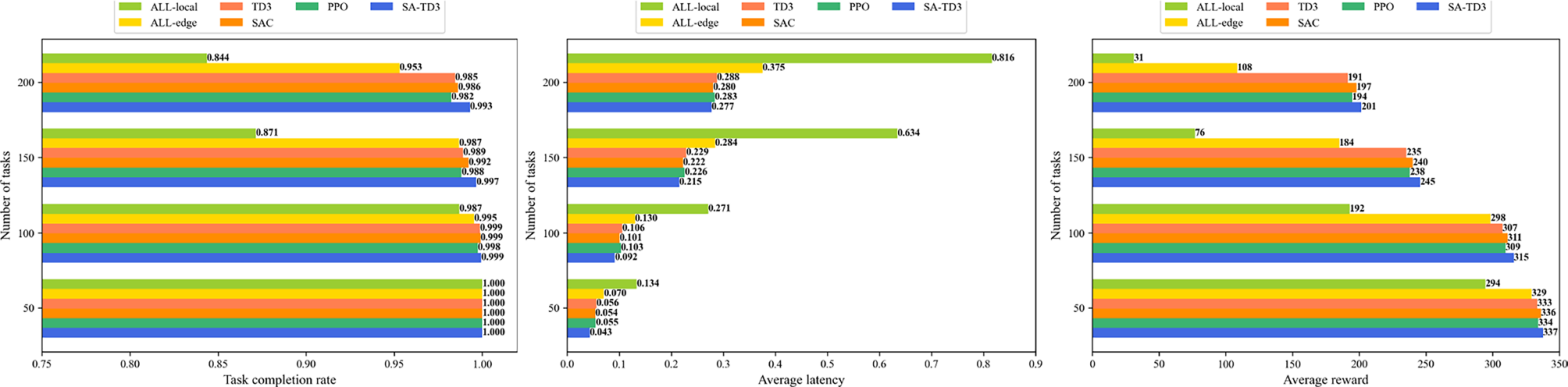
Task completion rate, average latency and reward under varying task loads.

Under varying task volumes, Fig. 7 illustrates algorithm performance, including task completion rate, average latency, and average reward. SA-TD3 consistently maintains high task completion, low latency, and high reward across all task loads, demonstrating strong scalability and robustness from small-scale to large-scale workloads.

ALL-local exhibits persistently poor performance due to rigid reliance on local resources. ALL-edge achieves better latency and reward for moderate loads but becomes unstable under extreme conditions, as over-dependence on UAVs introduces bottlenecks. Standard TD3 performs more effectively than both baselines but is still outperformed by SA-TD3, especially under high task loads where coordinated offloading and dynamic resource allocation are critical. Under rising task loads, SAC and PPO maintain stable completion rates and reasonable latency, with SAC slightly outperforming PPO in delay performance. However, the performance of these two methods in all three indicators was inferior to SA-TD3, especially under heavy load conditions.

Overall, SA-TD3 demonstrates superior adaptability to varying task volumes, effectively leveraging distributed resources and balancing loads among local nodes, UAVs, and idle nodes. Its high reward and low latency confirm its suitability for resource-constrained and large-scale MIoT scenarios.

In summary, across diverse evaluation scenarios, SA-TD3 achieves the highest task completion rate, lowest latency, and greatest average reward compared to ALL-local, ALL-edge, and standard TD3. Its integration of simulated annealing enables effective global exploration, while the dynamic priority replay mechanism enhances learning from critical experiences. These advantages make SA-TD3 a scalable solution for computation offloading and resource optimization in dynamic MIoT environments.

## Conclusion

The article proposes a hybrid decision-making framework, SA-TD3, to address the computational overload issues of maritime surface nodes in MIoT. The framework optimizes from the perspective of maritime surface nodes, enhances TD3 with simulated annealing and an environment-aware dual-channel advantage function, and introduces a GNN-based dynamic prioritized replay mechanism, enabling more efficient and robust offloading decisions. Simulation results demonstrate that the proposed method significantly reduces average latency and computation load, optimizes energy efficiency, and improves overall system performance compared with ALL-local, ALL-edge, and standard TD3 baselines. The SA-TD3 shows particular advantages in handling large-scale maritime tasks and complex environments, confirming its robustness and practical applicability. In the future, we will focus on model tuning, large-scale deployment, and the exploration of real-world applications, providing theoretical support and practical guidance for resource optimization in complex maritime environments. Moreover, we will extend the evaluation toward larger-scale maritime networks and multi-UAV collaborations to further validate the scalability of the proposed framework.

## Data Availability

The code and datasets analysed during the current study are not publicly available due to the ongoing nature of the associated research project, but are available from the corresponding author on reasonable request.
